# Wear Resistance of the Refractory WC–Co Diamond-Reinforced Composite with Zirconia Additive

**DOI:** 10.3390/ma18091965

**Published:** 2025-04-25

**Authors:** Boranbay Ratov, Volodymyr Mechnik, Edvin Hevorkian, Miroslaw Rucki, Daniel Pieniak, Nikolai Bondarenko, Vasyl Kolodnitskyi, Sergii Starik, Viktor Bilorusets, Volodymyr Chishkala, Perizat Sundetova, Aldabergen Bektilevov, Anar Shukmanova, Askar Seidaliyev

**Affiliations:** 1 Department of Geophysics and Seismology, Institute of Geology and Oil and Gas K. Turysova, Satbayev University, Satpaev Str. 22, Almaty 050013, Kazakhstan; 2 V. Bakul Institute for Superhard Materials, National Academy of Science of Ukraine, Avtozavodska Str. 2, 04074 Kyiv, Ukraine; 3 Faculty of Mechanical Engineering, Casimir Pulaski Radom University, Stasieckiego Str. 54, 26-600 Radom, Poland; 4 Institute of Mechanical Science, Vilnius Gediminas Technical University, Sauletekio al. 11, LT-10223 Vilnius, Lithuania; 5 Institute of Sustainable Technologies, Lukasiewicz Research Network, Pułaskiego 6/10, 26-600 Radom, Poland; 6 School of Physics and Technology, V.N. Karazin Kharkiv National University, 4 Svobody Sq., 61022 Kharkiv, Ukraine; 7 Faculty of Engineering, Caspian University of Technology and Engineering Named After Sh. Yessenov, 32 mkr., Aktau 130000, Kazakhstan; 8 Faculty of Transport Engineering and Logistics, Satbayev University, Satpayev St. 22A, Almaty 050013, Kazakhstan; 9 Department of Petroleum Engineering, Satbayev University, Satpaev Str. 22A, Almaty 050013, Kazakhstan

**Keywords:** mining, rock cutting, tungsten carbide, diamond, composites

## Abstract

This paper provides deeper insights into the performance of diamond particulate reinforced refractory composites used for cutting tools in the oil and gas industries. In particular, 25C_diamond_–70.5WC–4.5Co composites were enhanced with zirconia additives in proportions of 4 wt.% and 10 wt.% via the spark plasma sintering method. Wear tests were performed, and the analyses of elemental composition, morphology, and microstructure were completed. It was found that the addition of yttria-stabilized zirconia increased the plasticity of the matrix and thus introduced the ductile fracture mechanism, reducing the role of abrasive wear. As a result, the specific wear rate was reduced by 44% after the addition of 4 wt.% of zirconia and by 80% with 10 wt.% of ZrO_2_. The presence of zirconia contributed to the increase in the retention force between the matrix and diamond grits, which further reduced the intensity of the abrasive mechanism.

## 1. Introduction

WC–Co-based Polycrystalline Diamond Compacts are widely used in the petroleum and gas industries for rock drilling and the machining of non-ferrous materials [1]. These composites exhibit high strength, hardness, and wear resistance combined with high density and thermal conductivity [2,3]. The properties of the composite depend on the size and morphology of the original powders, including diamond grain size and the phase composition and microstructure of the material, as well as on the methods and parameters of the sintering process [4,5,6]. During the drilling through the hard, abrasive rocks, the refractory matrices of cutting inserts undergo intensive abrasive, fatigue, and adhesive wear under harsh work conditions, which shortens their working life and poses limitations upon their applications. In particular, Chen et al. [7] demonstrated a decrease in the friction coefficient for diamond/POM pairs sintered at higher temperatures, exhibiting a combination of abrasive and adhesive wear. It was also demonstrated that the cutting tools under an alternating load of interrupted cutting at a speed of 580 m/min were unable to withstand the limit value of 6000 shocks [8]. Moreover, weak adhesion between the diamond grits and the matrix and the insufficient retention capability of the matrix usually led to grit pullout during and subsequent tool breakdown, as shown in a comprehensive review [9]. In particular, Han et al. applied nano-VN in order to enhance the holding force between the matrix and diamond [10].

The phenomenon of graphitization of the diamond grits and intense WC grain growth during sintering worsens the mechanical characteristics and performance of diamond-reinforced composites. For instance, Larindo et al. achieved a reduction of graphitization and oxidation in hybrid matrix composites (Mo, MoC, and Mo_2_C) sintered in a diamond-Mo system [11]. Chen et al. published a report [12] demonstrating that high-temperature sintering under high pressure allowed for the avoidance of diamond graphitization in TiC–diamond compacts, because the graphite reacted preferentially with titanium. Yin et al. experimented with grain growth inhibitors and found that a VC additive caused finer grain size and smaller size distribution in WC–6 wt.% Co cemented carbides, with an increasing Cr_3_C_2_/VC proportion decreasing the grain size [13].

It is also important to note that the brittleness of the refractory matrix may contribute to the destruction of the drill bit when cutting through hard rocks. Ponomarev et al. found that the free carbon formed at the carbide–binder interface decreased the strength and the wear-resistance of WC-based cemented carbides due to microcrack propagation along the carbide–binder interface [14]. Thus, the same authors undertook efforts to reduce the average concentration of carbon, slowing the carbide–binder interface reaction and carbon diffusion at high cooling rates [15].

The abovementioned challenges have motivated numerous researchers to undertake efforts to develop new ways of enhancing diamond-reinforced composites and increasing their efficiency. Among the propositions to solve the abovementioned problems, the application of spark plasma sintering (SPS), also known as the field-assisted sintering technique (FAST), is very popular due to its numerous advantages [16]. Tokita illustrated in a comprehensive review [17] that SPS made it possible to obtain composites with grain sizes close to the initial powder particle dimensions, hence, improving the mechanical properties. Su et al. [18] reported improvements in the wear resistance of WC–8Co cemented carbide due to the grain refinement promoted by a nano-alumina additive during SPS sintering. Zhao et al. [19] published a report on the preparation of near-nanocrystalline WC–10Co compounds by SPS using sub-micron WC powder and a nano-Co raw binder. In SPS methods, the respective portion of powder undergoes uniaxial pressure and simultaneous heating via the electrothermal effect in a vacuum or protective atmosphere. The main merits of SPS techniques are the high heating rate, which prevents grain growth, and diamond graphitization in the case of diamond-reinforced composites. A vacuum or protective atmosphere prevents the components from oxidizing.

Another way of improving sintered WC-based composites is by adding zirconia ZrO_2_. Yttria-stabilized tetragonal zirconia is one of the most widely applied ceramic materials due to its excellent performance at high temperatures, low thermal conductivity, very high hardness, fracture toughness, and good wear resistance [20]. For instance, Choi et al. prepared yttria-stabilized zirconia YSZ-10/20 vol% WC composite ceramics using pressureless sintering and hot pressing [21]. They noted an increase of fracture toughness and wear resistance of the composites. When added to ceramic composites, zirconia enhanced their performance due to its well-known transformation toughening mechanism [22]. When zirconia was added in form of a nanopowder, its amount could be reduced due to the positive impact of the SPS process in terms of retaining submicron grain sizes [23].

The present study is a continuation of previous research, which has demonstrated that SPS processing of C_diamond_–(WC–6 wt.%Co) composites with the addition of zirconia in proportions of 4 wt.% and 10 wt.% both promotes the formation of fine-dispersed structures with very thin binding layers of metallic phase between carbide grains and enhances the interface strength between the diamond grits and refractory matrix [24]. It was found that the addition of yttria-stabilized zirconia increased parameters such as indexes of elastic strain to failure, resistance to plastic deformation, and tolerance to abrasion damage [25]. The investigations presented in this paper were aimed to experimentally check the wear characteristics of C_diamond_–(WC–6 wt.%Co) composites with zirconia additive in correlation with the previous findings. To the best of our knowledge, this has not been reported so far. Thus, in this paper, we present the results of complex investigations of the structural characteristics of the composite around diamond grits and its wear resistance in the rock cutting process in relation to its zirconia content.

## 2. Materials and Methods

Samples of diamond–WC–Co-based cemented carbide composites were prepared using diamond powder C_diamond_, tungsten carbide WC, cobalt binder Co, and zirconia ZrO_2_. The diamond powder used was of grit size 500/400, produced by De Beers (Johannesburg, South Africa). Its particles had an average diameter of 0.45 mm. The tungsten carbide powder WC 1750H was made by Global Tungsten & Powders (Bruntal, Czech Republic), with an average particle size range of 2.0 μm to 8.0 μm. The particle size of the cobalt powder used in the samples for the binder was ca. 2.0–3.0 μm, and it was made by Kuybyshevburmash (Samara, Russia). The yttria-stabilized zirconia nanopowder (3 wt.% Y_2_O_3_) additive was produced by NANOE (Ballainvilliers, France). The particles had dimensions of ca. 100 nm.

The preparation procedure of the powder mixtures was similar to the one described in detail in [25]. Briefly, the sample without ZrO_2_ was prepared by adding moistened diamond powder to a mixture of WC and Co powders and then mixing the blend in an ethanolic medium. In turn, for the samples with zirconia additive, first ZrO_2_ and Co powders were mixed in an alcohol medium, and then an appropriate amount of WC was added. As a result, the following proportions by mass were obtained: 25C_diamond_–70.5WC–4.5Co (sample #1), 25C_diamond_–66.74WC–4.26Co–4ZrO_2_ (sample #2), and 25C_diamond_–61.1WC–3.9Co–10ZrO_2_ (sample #3). The specimens of diameter 25 mm and height 5 mm were sintered in vacuum using an electroconsolidation method, described in detail elsewhere [26]. In this method, a direct current of 5000 A generated heat by passing through the graphite mold and the powder. The method can be classified as a type of hot pressing (HP), since pressure and heat in electroconsolidation are applied to the initial powder simultaneously, like in traditional HP techniques [27]. Based on experimental data from previous research [28], a sintering temperature of 1350 °C, an uniaxial pressure of 30 MPa, and a holding time of 3 min. were chosen. The specimens then underwent grinding, reducing their height to 4.84 mm and diameter to 24.62 mm, and then polishing to achieve a mirror-like surface for further analysis.

In order to investigate the elemental composition, morphology, and microstructure of the sintered material, a Zeiss EVO 50 XVP (Zeiss, Oberkochen, Germany) scanning electron microscope (SEM), JEOL, Ltd., Tokyo, Japan was used, equipped with Energy Dispersive Spectrometry (EDS) unit type Ultim Max 100, manufactured by Oxford Instruments (High Wycombe, UK). In order to determine the grain boundaries and calculate their sizes with area distributions, ImageJ software https://imagej.net/ij/download.html (accessed on 10 April 2025) was employed [29].

**Figure 1:**
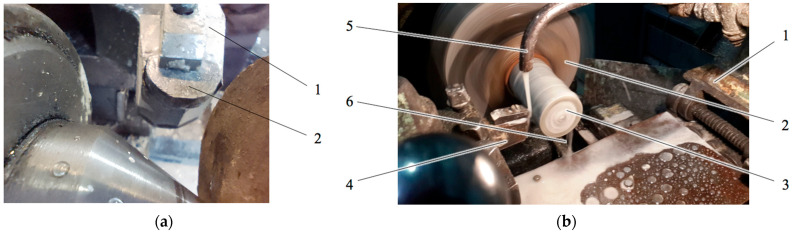
Rig for testing the wear resistance of the composite: (**a**) Fixation of the tested specimen; (**b**) Testing process.

From the numerous available wear tests [30,31,32], the one imitating the real conditions of rock cutting was selected. The wear resistance of the specimens was tested using a test rig based on a typical cutting tool, as shown in Figure 1. The specimen of diamond composite (denoted as 2 in Figure 1a) was fixed in a holder (1), and then the granite cylinder was turned. The granite was produced by Korostyshev Granite Plant (Korostyshev, Zhytomyr region, Ukraine) and belonged to the 10th drillability category. The rotational speed during the test was 400 rpm, and the cutting depth per one path was 0.5 mm, with a perpendicular feed of 0.5 mm/rotation and a longitudinal feed of 0.13 mm/rotation. The normal load was 10 N. The initial diameter of the turned cylinder was 70 mm, and the length of the turned part was 200 mm. Figure 1b illustrates the turning process, where (1) denotes the main bed, (2) shows the fixation of the granite cylinder (3), (4) is the composite specimen holder, (5) is the coolant feeder, and (6) is the waste sludge.

The weight loss of each tested diamond composite specimen was measured after 10 paths. This took ca. 3300 s, since a single path of 200 mm required 230 s. The worn surface of the tested specimens was investigated with an Axioscope 5 optical microscope (Zeiss, Germany).

From the data on the mass loss of the tested specimens, the wear rate was calculated according to the following equations, either in volumetric or mass categories [33]:

(1) $$W_{R}=\frac{\Delta W}{L},$$

(2) $$W_{V}=\frac{\Delta V}{t},$$

where *W_R_* is the mass wear rate, Δ*W* is the mass loss along the friction path *L*, *W_V_* is the volumetric wear rate, and Δ*V* is the volumetric loss during test time *t*. The friction path *L* for Equation (1) was calculated from the following simple relation:

*L* = *πDnt*, (3)

where *D* is the diameter and *n* is the number of rotations.

Then, specific wear rate *W_S_* was calculated as the volumetric loss per friction path length under normal loading force *F*, as follows [34]:

(4) $$W_{S}=\frac{\Delta V}{LF}$$

The masses of the specimens before and after the wear test were measured using an AD200 laboratory balance produced by AXIS Sp. z o.o. (Gdansk, Poland).

## 3. Results and Discussion

The results will be presented and discussed in subsections concerning the analysis of the initial powders, sintered composite microstructures, wear resistance results, and worn surfaces of the tested composites.

### 3.1. Analysis of the Initial Powders

**Figure 2:**
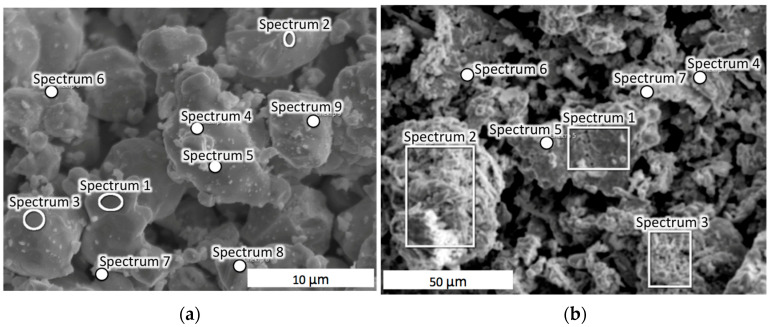
SEM images of the initial powders: (**a**) WC; (**b**) Co. The locations of EDS samplings are indicated.

Since the sinterability of ceramic powders is dependent on particle size and morphology, the distribution of the particle dimensions, and their degree of agglomeration [35], the initial powders underwent a thorough analysis. Figure 2 presents SEM images of the tungsten carbide (Figure 2a) and cobalt (Figure 2b) powders. The locations of the EDS analyses are indicated in Figure 2, while the respective results are collected in Table 1 and Table 2.

It can be seen from Figure 2 that the particles of both powders constituted agglomerates with dimensions above 10 μm. The results of our EDS analysis demonstrated that the ratio of tungsten to carbon was close to stoichiometric. In some locations, only pure tungsten was found, e.g., in Spectrum 3, recorded in Table 1.

In turn, the cobalt powder contained some oxygen and iron (Table 2), which suggested that some oxidation processes had occurred on the surface of the cobalt particles. Despite the presence of iron oxide contamination, the percentage of all oxides did not exceed 1 wt.%, so the initial cobalt powder could be considered pure. The oxidation of a metal in contact with air and humidity is a common occurrence. In general, elemental distribution was quite uniform and did not reveal any sort of strong chemical bonds between the particles before sintering.

**Figure 3:**
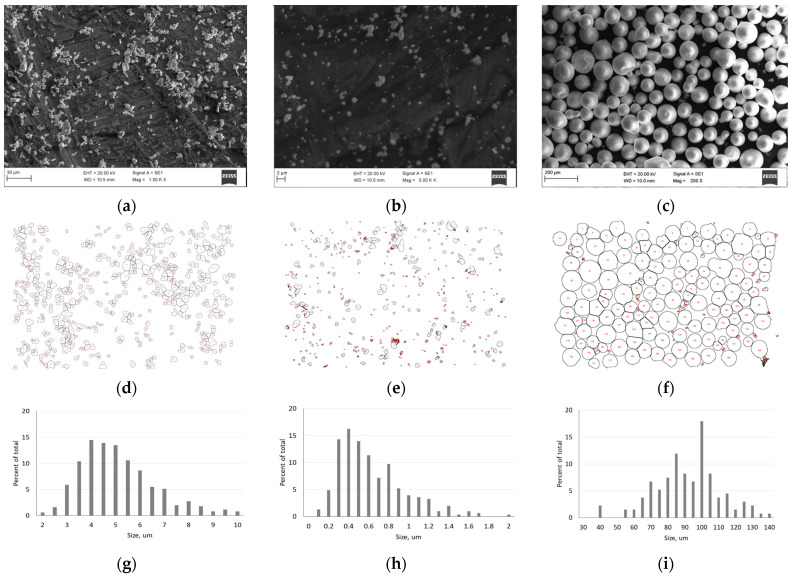
Morphology analysis of the initial powders: (**a**) SEM images of Co powder; (**b**) SEM images of WC powder; (**c**) SEM images of ZrO_2_ powder; (**d**) Dimensional measurement of Co powder; (**e**) Dimensional measurement of WC powder; (**f**) Dimensional measurement of ZrO_2_ powder; (**g**) Distribution of dimensions of Co particles; (**h**) Distribution of dimensions of WC particles; (**i**) Distribution of dimensions of ZrO_2_ particles.

The morphology of the powder particles is presented in Figure 3. There are SEM images of cobalt, tungsten carbide, and zirconia powders in Figure 3a–c, the results of particle diameter measurements in Figure 3d–f, and a diagram of the distribution of dimensions in Figure 3g–i.

The initial cobalt powder shown in Figure 3a,d,g consisted of non-uniformly shaped particles of dimensions between 3 µm and 8 µm but predominantly 4 µm. Some particles exhibited round shapes with metal build-up. Importantly, rounded particle shapes promote powder compaction in bulk state.

The tungsten carbide powder (Figure 3b,e,h) consisted of particles of 0.2–1.2 µm with a dominant dimension of 0.4 µm and a very small fraction of particles up to 2 µm. Their shapes were irregular, resembling cubic, right, and oblique prisms with dense structures; build-ups can be seen on their surfaces.

The zirconia powder seen in Figure 3c represents agglomerates of nanoparticles of average dimensions of 100 nm, as described in [24]. The agglomerates exhibited mostly spherical shapes of dimensions between 50 µm and 140 µm, i.e., predominantly 100 µm with a small fraction at 40 µm (Figure 3f,i). When added to the main batch, the agglomerates did not retain their form, allowing zirconia to be distributed uniformly in the powder blend; otherwise, agglomerates would promote pore formation during sintering, preventing the material from reaching high relative density [36]. Similar to chemical bonds, the initial powders did not reveal strong physical interrelations that would prevent the sintering of a repeatable, uniform structure of the composite.

### 3.2. Sintered Composite Microstructures

**Figure 4:**
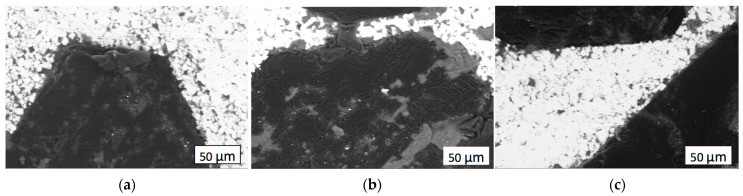
SEM images, with material contrast, of the sintered composites: (**a**) Sample 1 (25 wt.%C_diamond_–70.5 wt.%WC–4.5 wt%Co); (**b**) Sample 2 (25C_diamond_–66.74WC–4.26Co–4ZrO_2_); (**c**) Sample 3 (25C_diamond_–61.1WC–3.9Co–10ZrO_2_).

After sintering, all the samples with different proportions of components exhibited noticeable, easily definable boundaries between WC and Co grains. In the optical images, cobalt had a lighter color, while WC grains were darker. Examples of the obtained SEM images are presented in Figure 4. In the sample sintered without zirconia addition (shown in Figure 4a), pores may be seen both on the boundary between the diamond grit and matrix and on the matrix surface elsewhere. A lack of pores at the boundaries between the diamond and matrices with 4 wt.% and 10 wt.% zirconia (Figure 4b,c) suggested that the adhesion between the matrix and the diamond surface had been improved. Improvement of the holding strength of diamond composites is an important goal of many research teams. For instance, the addition of basalt fiber particles to Cu-based composites increased the holding strength by 10% [37], while a Mo_2_C coating improved the force holding diamonds through decreased contact area with Fe-Ni-WC-based matrices [38].

**Figure 5:**
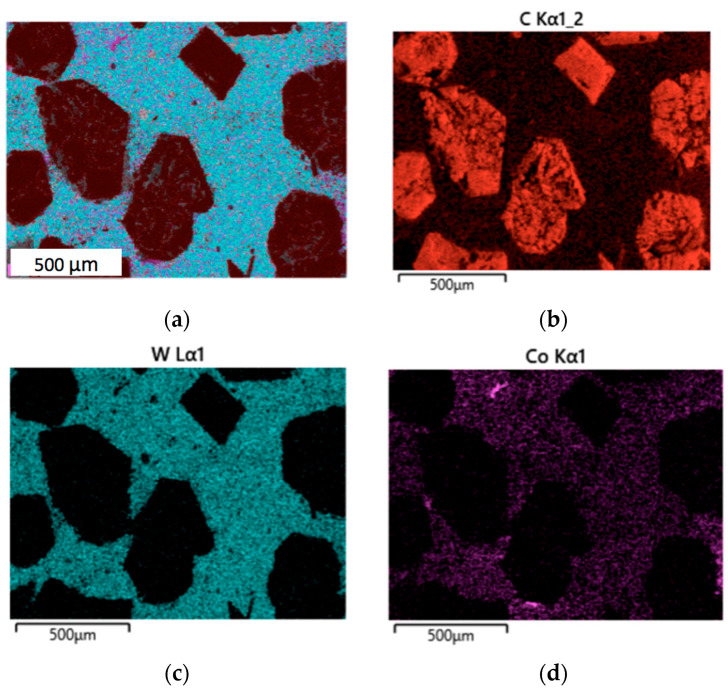
Results of an EDS analysis of the sintered composite 25 wt.%C_diamond_–70.5 wt.%WC–4.5 wt%Co with no zirconia additive: (**a**) Combined elemental map; (**b**) Distribution of carbon; (**c**) Distribution of tungsten; (**d**) Distribution of cobalt.

Elemental mapping was performed using the EDS method in order to analyze the distribution of the components on the surfaces of sintered specimens. Figure 5a presents a combined map of all components on the surface of the composite without zirconia, while Figure 5b–d illustrate the distribution of carbon, tungsten, and cobalt, respectively.

The maps of tungsten and cobalt distribution in Figure 5c,d reveal some irregularities in the elemental concentration on the surface; this could be attributed to the presence of diamond reinforcing particles in the refractory matrix. The diamond grits are well seen in Figure 5b, since carbon was mostly concentrated in diamond.

**Figure 6:**
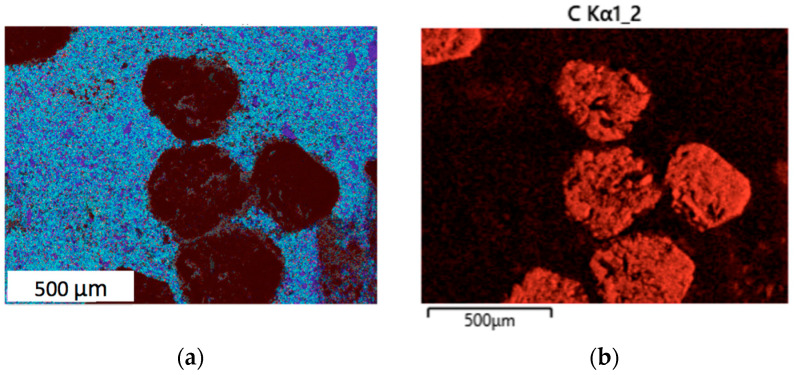

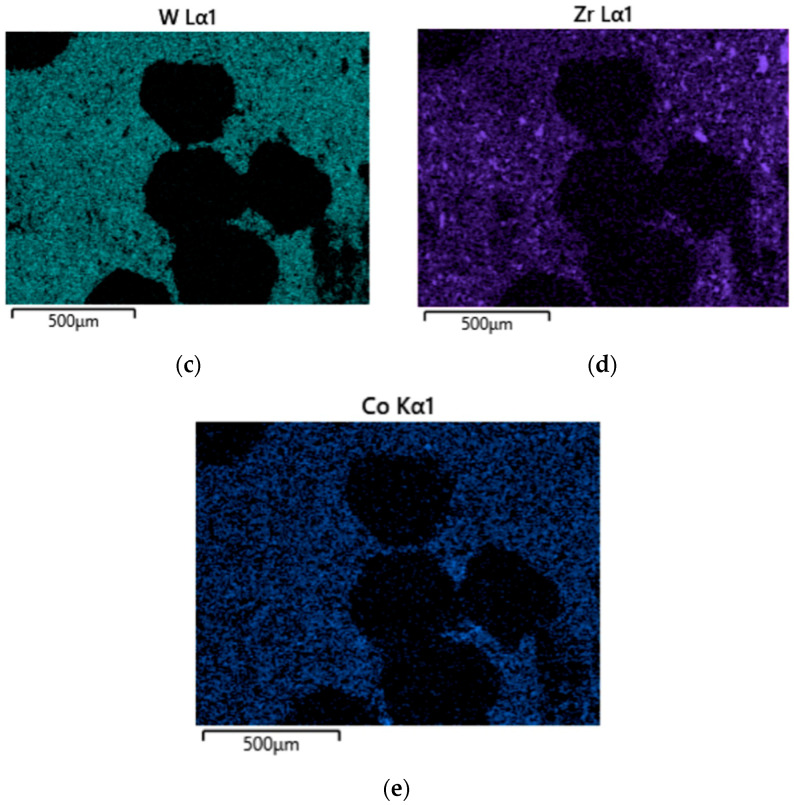
Results of EDS analysis of the sintered composite 25C_diamond_–66.74WC–4.26Co–4ZrO_2_: (**a**) Combined elemental map; (**b**) Distribution of carbon; (**c**) Distribution of tungsten; (**d**) Distribution of zirconium; (**e**) Distribution of cobalt.

The presence of zirconia additive in samples 2 and 3 contributed to a more uniform distribution of the components in the sintered specimens; this is illustrated in Figure 6, representing an elemental map of sample 2 with 4 wt.% of zirconia (25C_diamond_–66.74WC–4.26Co–4ZrO_2_). Notably, tungsten and cobalt were distributed uniformly (Figure 6b,c, respectively); this was distinguishable, even if negligible variations could be attributed to the surface topology. Thus, it can be concluded that the matrices with zirconia additive exhibited a more uniform distribution of tungsten carbide and cobalt phases.

Improved uniformity of the elemental distribution in the refractory matrix has been proven to be advantageous for a composite [39]. Especially in the case of nanopowders, evenly distributed phases can provide improved sintering properties and result in high-quality composite ceramics [40]. The preservation of small grain sizes in the WC–Co–ZrO_2_ matrices could be attributed to the peculiarities of the SPS technique, e.g., relatively low sintering temperatures and short holding times, among other factors.

### 3.3. Wear Resistance Results

The abovementioned improved structural features of the WC–Co–diamond composites with zirconia additive suggested possible enhancement of the related material characteristics, in particular, its wear resistance. Indeed, the wear tests performed according to the procedure described in Section 2 indicated significant improvement after zirconia addition to the matrix. Table 3 presents the wear rate results of the tested proportions, i.e., with no zirconia, with 4 wt.%, and with 10 wt.% of zirconia. Each result is represented by the arithmetic mean value from three experiments.

The specific wear rate of order 10^−7^ mm^3^/(N∙m) corresponded to that of anti-wear material and is typically achieved for WC–Co composites [41]. This was more advantageous than that of diamond-reinforced copper-beryllium alloy, which exhibited a specific wear rate of 6.35 × 10^−5^ mm^3^/(N∙m) [42]. The achieved wear rate for sample 3 in Table 3 was also better than the best result of 2.31 × 10^−7^ mm^3^/(N∙m), recently reported for WC–Co–GO (graphene oxide) cemented carbide [43].

It may be seen in Table 3 that compared to the sample 1, the wear rate by mass decreased by 47% after the addition of 4 wt.% of zirconia, and by 82% for 10 wt.% of added zirconia. A similar trend can be seen in the wear rate by volume and the specific wear rate. The improved wear resistance can be explained by the presence of monoclinic phase *m*-ZrO_2_, which contributed to the strengthening of the matrix and increasing its fracture toughness [25], as well as to the higher diamond holding force [23]. In the composite with a zirconia content of 10 wt.%, a larger percentage of tetragonal phase *t*-ZrO_2_ transformed into monoclinic *m*-ZrO_2_, contributing to a further significant improvement of wear resistance. A specific 80% wear rate reduction confirmed an improvement in the structural features related to the densely packed *m*-ZrO_2_ phase. Thus, the main factor determining the dramatic improvement in wear resistance was the percentage of zirconia nanopowder, combined with the morphology and size of the initial particles. Considering the demonstrated wear rates, it seems reasonable to apply a 25C_diamond_–61.1WC–3.9Co–10ZrO_2_ composite in the design and manufacture of tools for drilling hard and abrasive rocks.

### 3.4. Worn Surfaces of the Tested Composites

**Figure 7:**
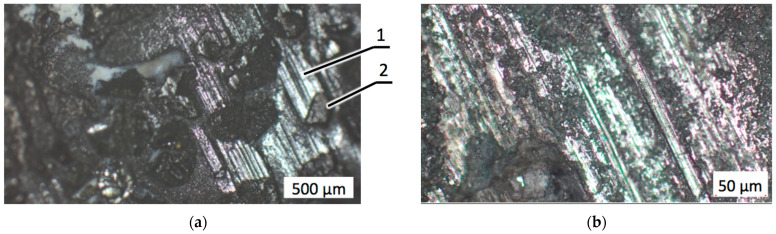
Optical microscopic images with different magnifications of the composite 25C_diamond_–70.5WC–4.5Co after the wear test: (**a**) Hundreds of microns; (**b**) Tens of microns. Trace (1) was left by the torn off diamond grit, trace (2) indicates the three-body damage mechanism.

During the wear tests, the diamond composite specimens underwent intense abrasion accompanied with falling off the diamond grits. When diamond grits are pulled off due to insufficient retention strength, the centers emptied of reinforcement particles weaken the drill bit, generating inner stresses in the material [44]. Figure 7 illustrates the worn surfaces of the diamond-reinforced composite 25C_diamond_–70.5WC–4.5Co with different magnifications.

An analysis of the worn surfaces shown in Figure 7 indicated that in the composite without zirconia addition, harsh wear conditions caused the loss of a significant quantity of diamond grit, leaving empty places throughout the worn surface. Deep and wide grooves and scratches appeared on the surface of the refractory matrix as a result of the abrasive wear caused by the granite counterpart (two-body wear) and by debris trapped between the two surfaces penetrating the soft cobalt phase (three-body wear). The presence of diamond grits torn out of the matrix significantly increased the intensity of three-body wear.

**Figure 8:**
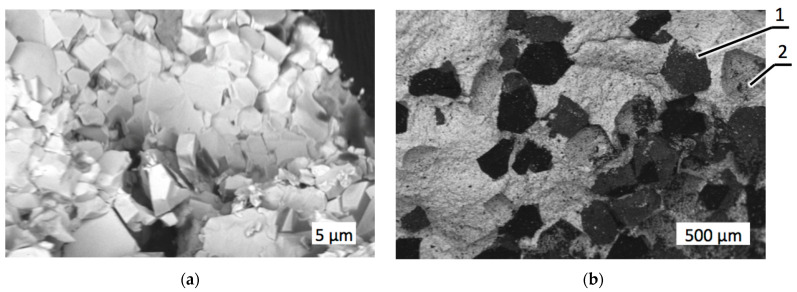
SEM images, with material contrast, of composite 25C_diamond_–70.5WC–4.5Co after the impact test: (**a**) Fracture area of the matrix; (**b**) Fracture area with diamond reinforcement. 1—Undamaged diamond, 2—After a whole diamond had been torn out.

It should be considered that in real mining conditions, impact load plays an important role in the wear of the drill bit and cutting edges. As shown in Figure 8, fracture under impact load took place along the grain boundaries of the matrix itself (Figure 8a) and the ones between the matrix and diamond grits (Figure 8b). In both cases, the grain boundaries appeared to be the weakest link in the chain, with features typical of a brittle intergranular fracture.

**Figure 9:**
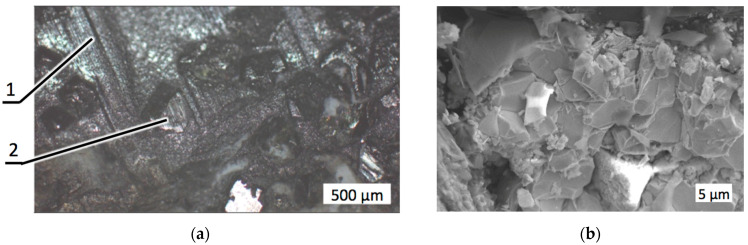
Images of composite 25C_diamond_–66.74WC–4.26Co–4ZrO_2_: (**a**) Optical image after the wear test; (**b**) SEM image after impact load. 1—abrasive wear trace, 2—worn surface of a diamond.

The addition of ZrO_2_ powder substantially improved the structural characteristics of the composite. Figure 9a presents an example of the worn surface of the composite with 4 wt.% of zirconia added to the matrix. Apart from the decreased number of diamond grits, it exhibited smoother and more evenly distributed scratches on the matrix surface. Evidently, the role of the three-body mechanism decreased the abrasive wear of specimens 25C_diamond_–66.74WC–4.26Co–4ZrO_2_ compared to that of 25C_diamond_–70.5WC–4.5Co.

The features of fracture surfaces shown in Figure 9b show differences compared to the characteristics of the fracture surface shown in Figure 8a, which belonged to the specimen with no zirconia added. In particular, some pits with structural dimensions of ca. 100–200 nm can be seen in the structure of the fracture surface in Figure 9b. The areas with pitted destruction indicated a ductile wear mechanism along with brittle wear, represented by typical chipping. The mixed wear mechanism contributed to the improvement of wear resistance, as indicated in Table 3 above.

**Figure 10:**
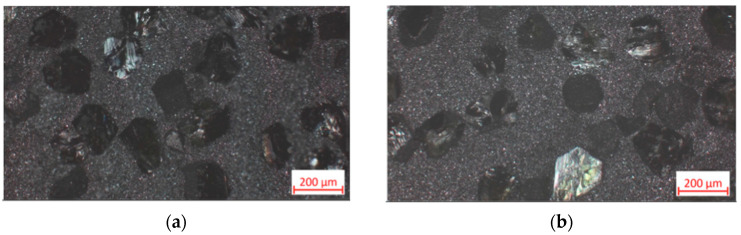
Examples of the optical microscopic images of the surface of the composite 25C_diamond_–61.1WC–3.9Co–10ZrO_2_ after the wear test: (**a**) Normal light; (**b**) Polarized light.

The increase of diamond holding capacity was assessed visually. It was observed that the samples with zirconia additives exhibited a simultaneous wear of the diamond and the matrices, with the diamonds having been worn and destroyed before they were torn away, as shown in Figure 9a. At the same time, holes in WC–Co matrix indicated that it was not able to hold the diamond grits, which fell off prematurely. A further increase of the zirconia proportion to 10 wt.% caused further enhancement of the diamond retention force in the refractory matrix. In the previously reported fracture tests, this composite exhibited a typical ductile damage mechanism of the matrix, and damaged diamond grits were still held in the matrix [45]. The wear tests confirmed the exceptional adhesion and clamping forces that kept the diamond in the refractory matrix after turning the granite cylinder. Moreover, the surfaces shown in Figure 10a,b exhibited no significant scratches related to the abrasive damage of the matrix. Obviously, the three-body wear mechanism was substantially reduced, if not eliminated, in the case of 25C_diamond_–61.1WC–3.9Co–10ZrO_2_ specimen.

**Figure 11:**
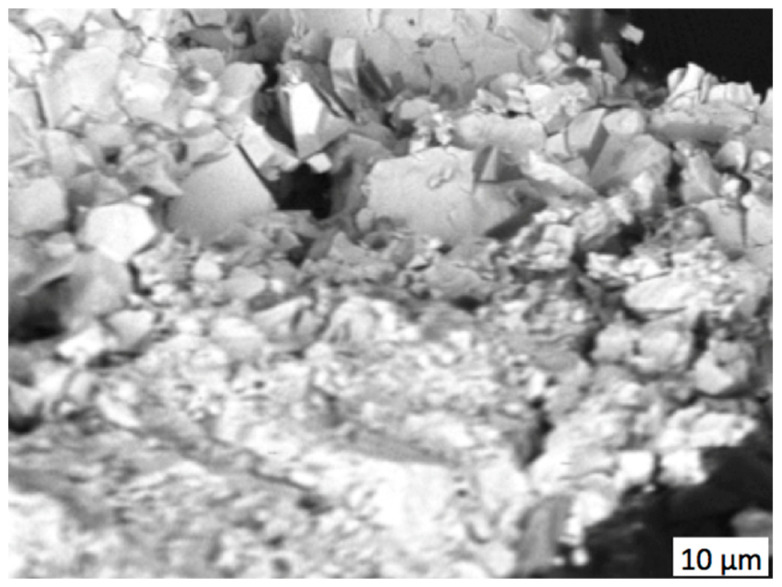
SEM image (material contrast) of the fracture surface of the composite 25C_diamond_–61.1WC–3.9Co–10ZrO_2_.

Thus, experimental results confirmed the enhancing effect of zirconia addition to the diamond-reinforced WC–Co matrix sintered using the electroconsolidation method. The addition of 4 wt.% substantially reduced abrasion wear intensity, and the addition of 10 wt.% zirconia minimized the three-body wear mechanism due to the strong retention of the diamond grits. Moreover, the mechanism of fracture of the composite altered after the content of zirconia was increased to 10 wt.%. Figure 11 presents a fracture surface with a highly developed network of microcracks, typical for transgranular fractures with a cleavage of facets.

Fractography is essential for understanding the failure mechanisms, especially when interfaces play an important role. In particular, the fractured surface of the zirconia bend bar exhibited grains of angulated shape as a result of intergranular or transgranular fractures [46]. Intergranular fractures dominated in the WC–Co diamond-reinforced composite with strain concentrations along the grain boundaries and martix–diamond interfaces, which caused decohesion between adjacent grains and between the matrix and diamond grits. Predominantly intergranular damage can be concluded from the SEM image in Figure 8a, where many grapes of similar shapes and sizes are seen, while in Figure 11, many smaller, crushed grains are seen along with larger ones.

The grain fragmentation seen in Figure 11 proved that the zirconia additive promoted enhanced grain boundaries. Multiple crack branching and the transglanular fracture mechanism contributed to increased fracture toughness and wear resistance. Stresses concentrated around the diamond grit resulted from strong retention forces, which caused transgranular destruction of the diamond reinforcement rather than its removal from the matrix.

It was reported previously [23] that in a WC–Co matrix with no zirconia added, large cobalt areas form, as well as direct contact surfaces between the tungsten carbide grains. The addition of zirconia nanopowder promoted formation of thin layers of cobalt of ca. 100 nm between the WC grains and worked as a grain growth inhibitor, which resulted in improved plasticity and fracture toughness, even though the hardness somewhat decreased. In particular, the addition of 6 wt.% zirconia increased the fracture toughness of the 94WC–6Co matrix composite from *K*_Ic_ = 15.7 up to 16.9 ± 0.76 MPa·m^0.5^.

The results of the present research confirm the trend exhibited by the index of tolerance to abrasion damage 1/(*E*^2^*H*). This index is used for the prediction of the resistance of tested materials to abrasive wear on the basis of their mechanical characteristics, i.e., *H* hardness and *E* modulus [47]. It was demonstrated in [25] that the index 1/(*E*^2^*H*) calculated for the WC–Co matrix increased twofold from 0.75 × 10^−7^ up to 1.5 × 10^−7^ GPa^−3^ after 4 wt.% of zirconia was added and rose further to 2.75 × 10^−7^ GPa^−3^ with an increase in the zirconia content up to 10 wt.%. These results corresponded to the measured decrease of wear rate from 7.6 × 10^−13^ m^3^/(N·m) down to 4.3 and 1.6 for the respective ZrO_2_ proportions, as specified in Table 3. Therefore, it can be concluded that the wear resistance was improved due to the abovementioned zirconia-promoted structural features, leading to a denser matrix with enhanced diamond-retention forces and improved adhesion.

## 4. Conclusions

The experimental results confirmed an improvement of wear resistance of the 25C_diamond_–70.5WC–4.5Co composite after zirconia addition. The composites were sintered in vacuum via an electroconsolidation method with a holding time of 3 min at temperatures of 20–1350 °C under a mechanical pressure 30 MPa. The following conclusions can be drawn from the experimental results.

1. The fracture mechanism of the composite 25C_diamond_–70.5WC–4.5Co exhibited a mainly intergranular mechanism, including the interfaces between the matrix and diamond grits. The addition of 4 wt.% and 10 wt.% of zirconia introduced a ductile fracture mechanism due to the increased plasticity of the matrix. Multiple crack branching and transglanular fracture mechanisms contributed to the increased fracture toughness and wear resistance of the composites with the zirconia additive. Interfaces between the matrix and diamond reinforcement became denser and revealed no pores or defects.
2. The zirconia additive improved the wear resistance of the diamond-reinforced composite, reducing the specific wear rate by 44% with a content of 4 wt.% and by 80% with a content of 10 wt.% of ZrO_2_. This result is consistent with previously published reports indicating a substantial increase of the index of tolerance to abrasion damage 1/(*E*^2^*H*) after the addition of the respective proportions of zirconia.
3. In the composite with a zirconia content of 10 wt.%, a larger percentage of tetragonal phase *t*-ZrO_2_ transformed into the densely packed monoclinic one, i.e., *m*-ZrO_2_, contributing to the improvement of wear resistance.
4. In the tested composite without zirconia addition, the abrasive wear mechanism dominated, accompanied by the pullout of diamond grit from the matrix and, thus, increased three-body abrasive damage to the soft cobalt matrix. The zirconia additive contributed to the increase of the retention force and reduced the intensity of the abrasive mechanism. These improvements can be attributed to the dispersion hardening of the refractory matrix and subsequent stronger clasping of the diamond grits.

These results are important for the further design and fabrication of drill bits with diamond-reinforced composites. Further research will include impact tests and other experiments related to real work conditions of mining tools.

## Figures and Tables

**Table 1:** Results of EDS analysis of the WC powder in wt.%.

Spectrum	9	1	2	3	4	5	6	7	8
C, %	9.52	6.37	11.72	-	10.73	9.14	8.89	9.10	-
W, %	90.48	93.63	88.28	100.00	89.27	90.86	91.11	90.90	100.00
Total, %	100.00	100.00	100.00	100.00	100.00	100.00	100.00	100.00	100.00

**Table 2:** Results of EDS analysis of the Co powder (wt.%).

Spectrum	7	1	2	3	4	5	6
O, %	0.21	0.42	-	0.98	0.29	1.18	1.12
Fe, %	0.25	-	0.34	-	-	0.19	0.43
Co, %	99. 54	99.58	99.66	99.02	99.71	98.63	98.45
Total, %	100.00	100.00	100.00	100.00	100.00	100.00	100.00

**Table 3:** Results of the wear tests of the diamond composites.

Sample	Composition, wt.%	Wear Rate by Mass *W_R_*, 10^−5^ g/m	Wear Rate by Volume *W_V_*, 10^−12^ m^3^/s	Specific Wear Rate *W_S_*, 10^−13^ m^3^/(N·m)
1	25C_diamond_–70.5WC–4.5Co	9.380 ± 0.657	9.487 ± 0.759	7.552 ± 0.529
2	25C_diamond_–66.74WC–4.26Co–4 ZrO_2_	4.986 ± 0.399	4.470 ± 0.451	4.272 ± 0.299
3	25C_diamond_–61.1WC–3.9Co–10 ZrO_2_	1.751 ± 0.123	2.302 ± 0.138	1.571 ± 0.126

## Data Availability

Data are contained within the article.

## Abbreviations

The following abbreviations are used in this manuscript:

EDS	Energy Dispersive Spectrometry
FAST	Field-Assisted Sintering Technique
SEM	Scanning Electron Microscope
SPS	Spark Plasma Sintering
